# Impact of Body Mass Index on Pathological Response after Neoadjuvant Chemotherapy: Results from the I-SPY 2 trial

**DOI:** 10.21203/rs.3.rs-2588168/v1

**Published:** 2023-05-31

**Authors:** Haiyun Wang, Douglas Yee, David Potter, Patricia Jewett, Christina Yau, Heather Beckwith, Allison Watson, Nicholas O’Grady, Amy Wilson, Susie Brain, Paula Pohlmann, Anne Blaes

**Affiliations:** Cancer Care Associates; University of Minnesota Department of Medicine: University of Minnesota Twin Cities Department of Medicine; University of Minnesota Department of Medicine: University of Minnesota Twin Cities Department of Medicine; University of Minnesota Department of Medicine: University of Minnesota Twin Cities Department of Medicine; University of California San Francisco; University of Minnesota Department of Medicine: University of Minnesota Twin Cities Department of Medicine; Sanford Health; University of California San Francisco; Quantumleap; University of California San Francisco; MD Anderson Nellie B Connally Breast Center: The University of Texas MD Anderson Cancer Center Nellie B Connally Breast Center; University of Minnesota Medical Center

**Keywords:** Obesity, Body Mass Index, Breast Cancer, Neoadjuvant Chemotherapy, Pathological Complete Response

## Abstract

**Purpose:**

Increased body mass index (BMI) has been associated with poor outcomes in women with breast cancer. We evaluated the association between BMI and pathological complete response (pCR) in the I-SPY 2 trial.

**Methods:**

978 patientsenrolled in the I-SPY 2 trial 3/2010–11/2016 and had a recorded baseline BMI prior to treatment were included in the analysis. Tumor subtypes were defined by hormone receptor and HER2 status. Pretreatment BMI was categorized as obese (BMI≥30 kg/m2), overweight (25≤BMI < 30 kg/m2), and normal/underweight (< 25 kg/m2). pCR was defined as elimination of detectable invasive cancer in the breast and lymph nodes (ypT0/Tis and ypN0) at the time of surgery. Logistic regression analysis was used to determine associations between BMI and pCR. Event-free survival (EFS) and overall survival (OS) between different BMI categories were examined using Cox proportional hazards regression.

**Results:**

The median age in the study population was 49 years. pCR rates were 32.8% in normal/underweight, 31.4% in overweight, and 32.5% in obese patients. In univariable analysis, there was no significant difference in pCR with BMI. In multivariable analysis adjusted for race/ethnicity, age, menopausal status, breast cancer subtype, and clinical stage, there was no significant difference in pCR after neoadjuvant chemotherapy for obese compared with normal/underweight patients (OR = 1.1, 95% CI: 0.68–1.63, p = 0.83), and for overweight compared with normal/underweight (OR = 1, 95% CI: 0.64–1.47, p = 0.88). We tested for potential interaction between BMI and breast cancer subtype; however, the interaction was not significant in the multivariable model (p = 0.09). Multivariate Cox regression showed there was no difference in EFS (p = 0.81) or OS (p = 0.52) between obese, overweight, and normal/underweight breast cancer patients with a median follow-up time of 3.8 years.

**Conclusions:**

We found no difference in pCR rates by BMI with actual body weight based neoadjuvant chemotherapy in this biologically high-risk breast cancer population in the I-SPY2 trial.

## Introduction

Observational studies have shown increased body mass index (BMI) is a risk factor for developing breast cancer, especially hormone receptor positive breast cancers [1, 2]. Obesity and being overweight are also associated with advanced stage of breast cancer at diagnosis and have been independently associated with poor breast cancer outcomes [3–5]. Pathological complete response (pCR) is a surrogate of long-term outcomes of locally advanced breast cancer such as event-free survival (EFS) and overall survival (OS) [6, 7]. Studies investigating the relationship between BMI and pCR after neoadjuvant chemotherapy in breast cancer have demonstrated mixed results, with some revealing an association of increased BMI with poorer pCR rates after neoadjuvant chemotherapy [8–11], while others did not reveal any significant association [12–14]. Most were retrospective studies, some using data from more than a decade ago [8]. Chemotherapy regimens varied substantially from study to study, as did chemotherapy dosage. Since oncology clinical practices may cap chemotherapy dosage to a maximum body-surface area (BSA) of 2.0m2 to avoid increased toxicity [11, 15], it is not clear if the observed worse pCR rate in obese breast cancer patients is related to chemotherapy underdosing rather than BMI itself [8, 11]; and it is also unclear whether the observed worse pCR in obese patients has any correlation with breast cancer biological subtypes.

The I-SPY 2 (Investigation of Serial studies to Predict Your Therapeutic Response with Imaging and Molecular AnaLysis 2, NCT01042379) trial is an ongoing, multicenter, adaptive, phase II clinical trial platform that includes multiple experimental arms to evaluate new agents combined with standard neoadjuvant chemotherapy for the treatment of breast cancers with a high risk of recurrence, in comparison to standard chemotherapy regimen in a common control arm [16]. The trial uses pCR as primary end point. Importantly, chemotherapy dosage is not capped, but is given based on actual body weight [17]. The I-SPY 2 trial platform provides the advantage of eliminating some of the above-mentioned confounding factors while studying the association of BMI and neoadjuvant chemotherapy outcomes of breast cancer.

The purpose of this study was to examine the association of BMI with pCR, EFS, and OS in women with high-risk early stage breast cancer enrolled in the I-SPY 2 trial.

## Methods

### Study population and data collection

Women aged 18 years or older with a diagnosis of clinical stage II or III breast cancer, with a tumor diameter of at least 2.5cm by clinical examination and at least 2cm as assessed by imaging were eligible to participate in the I-SPY 2 trial. Exclusion criteria were an Eastern Cooperative Oncology Group performance status score greater than 1, and prior chemotherapy for this cancer. Patients with hormone receptor positive tumors and low risk MammaPrint^®^ scores were also excluded given the lack of benefit from systemic chemotherapy [18].

In this trial, participants were randomized to different neoadjuvant treatment regimens based on biomarker status, determined by Bayesian probabilities of pCR within a specific biomarker subtype with the experimental regimen. The biomarker status was based on hormone receptors (HR), human epidermal growth factor receptor 2 (HER2) and a 70-gene assay of MammaPrint^®^ at baseline. All participants received weekly intravenous paclitaxel (12 doses of 80mg per square meter of BSA) alone (control arm), or in combination with the assigned experimental regimen (experiment arm), followed by four doses of intravenous doxorubicin (60mg per square meter of BSA) and cyclophosphamide (600mg per square meter of BSA) every two to three weeks, with myeloid growth factor support if needed. Patients with HER2 + cancer also received trastuzumab for the first 12 weeks, given with a loading dose of 4mg per kilogram of body weight (week 1), followed by a maintenance dose of 2mg per kilogram every 3 weeks (weeks 4, 7, and 10). After receiving accelerated approval from the FDA [19], Pertuzumab was added to standard therapy for HER2 + patients, given with a loading dose of 840mg (week 1), followed by a maintenance dose of 420mg every 3 weeks (weeks 4, 7, and 10). All chemotherapy drugs were dosed based on actual body weight. Patients then underwent surgery which included axillary lymph node sampling. Radiation and adjuvant endocrine therapy after surgery were recommended in accordance with standard guidelines.

All participants provided written informed consent before undergoing screening for the study, and a second consent was obtained before treatment was initiated if the individual was eligible after random assignment to open-label treatment arms. All participating sites of this trial received approval from an institutional review board.

### Measures

The primary outcomes in this analysis were pCR [20], defined as elimination of detectable invasive cancer in the breast and lymph nodes (ypT0/Tis and ypN0) at the time of surgery; RCB (residual tumor burden) if pCR was not achieved [21]; and EFS and OS. The primary exposure of interest for this analysis was pretreatment BMI, categorized as obese (BMI≥30 kg/m2), overweight (25≤BMI < 30 kg/m2), and normal/underweight (< 25 kg/m2) based on World Health Organization criteria.

Demographic and clinical covariates included in multivariate analysis and defined a priori were age at screening (years); race/ethnicity (Non-Hispanic White vs. Non-Hispanic Black/African American vs. Latinx vs. other); breast cancer subtype including HR+/HER2+, HR+/HER2−, HR−/HER2 + and triple negative (HR−/HER2−); menopausal status (pre- vs. peri- vs. post-menopausal); and advanced vs. early tumor stage (stage III vs. I or II).

### Statistical analysis

Chi-squared and Anova were used to evaluate the association between BMI category and patient characteristics as appropriate. Logistic regression analysis was used to estimate associations between BMI and pCR, and linear regression to estimate the association between BMI and RCB; and Cox proportional hazards regression to estimate the associations between BMI and EFS, and between BMI and OS. These models were adjusted for the covariates listed above; because of limited degrees of freedom due to the total number of events, we excluded race/ethnicity from the covariates in the survival analyses. We report odds ratios (OR), linear coefficients, hazard ratios (HR), and respective 95% confidence intervals (CI). OR > 1 indicate greater odds of having pCR; hazard ratios > 1 indicate greater hazard of dying or having a major event. Analyses were run in SAS 9.4. All statistical tests were two-sided, and P values less than 0.05 were considered statistically significant.

## Results

### Patient characteristics

In total, 977 patients with a recorded baseline BMI were included in this study. Of these, 35.6% (N = 348) were normal/underweight, 31.6% (N = 309) overweight, and 32.8% (N = 320) obese (Table 1). The mean age was 48.7 ± 10.6 years. Overweight (mean age 49.9 years) and obese (mean age 49.7 years) patients were significantly older than normal/underweight patients (mean age 46.8; p < 0.0001). There were more Non-Hispanic Black / African American and Hispanic participants among those who were obese compared to normal/underweight and overweight. BMI category was not significantly associated with menopausal status, cancer stage, or cancer hormonal subtype.

### Relationship between BMI and Pathological Response

The overall pCR rate after neoadjuvant chemotherapy was 32.2%. pCR rates were 32.8% in normal/underweight, 31.4% in overweight, and 32.5% in obese patients, with no significant difference in the unadjusted or adjusted analysis (obese vs. normal/underweight, unadjusted OR = 0.99, 95% CI 0.71–1.37, adjusted OR = 1.05, 95% CI 0.68–1.63; overweight vs. normal/underweight, unadjusted OR = 0.94, 95% CI 0.68–1.30, adjusted OR = 0.97, 95% CI 0.64–1.47, Table 2). We ran an additional sensitivity analysis with continuous BMI as predictor, and this association was not significant, either.

Although an interaction between BMI and hormonal breast cancer subtype was not significant, we ran the unadjusted logistic regression models (predictor: continuous BMI) stratified by cancer hormonal subtype because cancer outcomes typically differ by hormonal subtypes. The association of BMI with PCR status was not significant in any of these models (Table 2). We did notice a trend towards decreased pCR rates with increasing BMI in the HR−/HER2 + subgroup (N = 88, Table 3) which did not reach statistical significance. The pCR rate in this hormonal subtype group was 75% in normal/underweight, 64.5% in overweight, and 48.3% in obese patients (overall p = 0.11). Using linear regression to compare Residual Cancer Burden (RCB) by BMI, RCB index was not associated with BMI category in either the unadjusted or the adjusted model, or for any cancer hormonal subtype after stratification (Table 4).

### Relationship of BMI with EFS and OS

**Figure 1:**
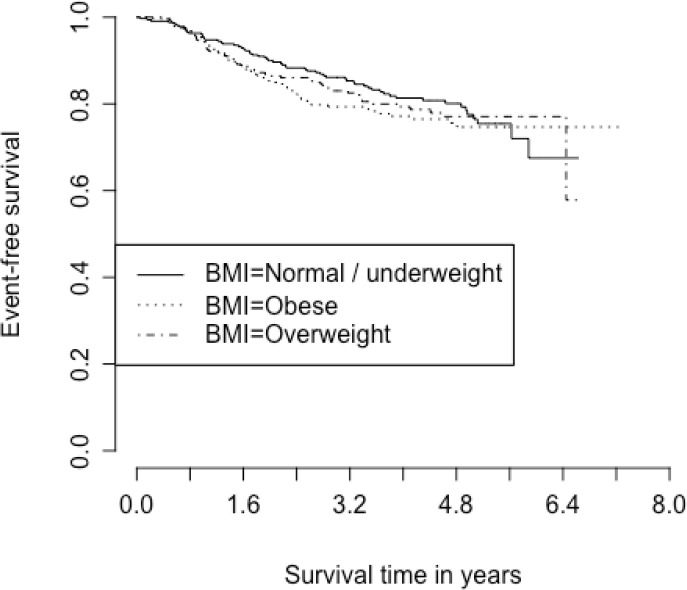
Kaplan Meier curve for event-free survival based on BMI category

**Figure 2:**
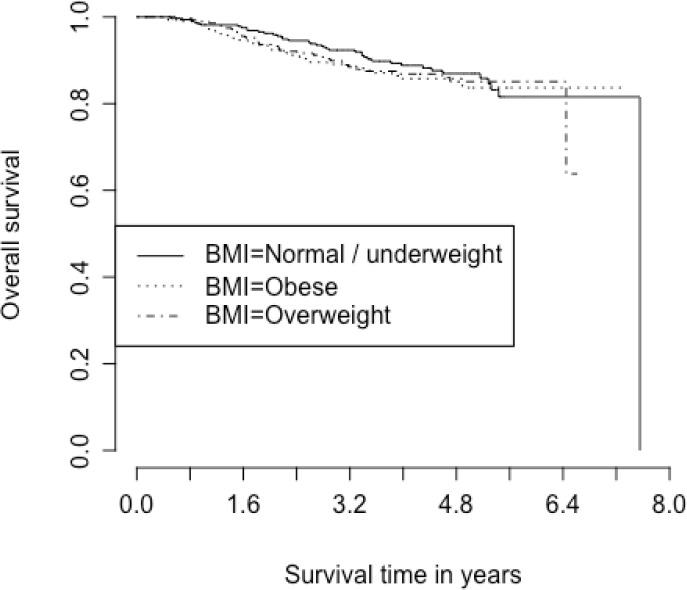
Kaplan Meier curve for overall survival based on BMI category

With a median follow-up time of 3.8 years, estimated OS at 5 years was 85.3% (95% CI 82.3–87.8%; 111 deaths out of 895 participants with known survival status) and estimated EFS at 5 years was 76.5% (95% CI 73.1–79.5%; 182 events out of 895 participants with known event status). BMI was not associated with EFS or OS in this study population (Tables 5 and 6). Due to limited events, we were unable to stratify these models by cancer hormonal subtype. Kaplan Meier curves for EFS and OS in different BMI categories are shown in Figs. 1 and 2.

## Discussion

In this clinical trial using actual body weight-based chemotherapy, higher baseline BMI was not associated with decreasing pCR rate after neoadjuvant chemotherapy in biologically high-risk early stage breast cancer patients, nor was it associated with worse EFS or OS. The overall pCR rate was 32.2% in our study, which was modest in comparison of other studies [22, 11]. The I-SPY 2 trial used standard chemotherapy regimen +/− HER2 targeted therapy depending on the HER2 status. It should also be noted, however, that this clinical trial also included patients with HR + breast cancer which have historically demonstrated lower response rates to chemotherapy [16]. This may explain why the overall pCR rate was modest after including the HR+/HER2− population, as HER2 + patients had a considerably higher pCR rate of 68% in our study [20].

Although several prospective studies and meta-analyses have reported that increased body weight was associated with poorer breast cancer outcomes such as OS and EFS, especially in postmenopausal women [23, 15, 24], it has been a challenge to clarify the underlying cause. In part, this has been attributed to possible interactions between BMI and comorbidities such as diabetes, coronary artery disease, cerebral artery disease, and socioeconomic status [25–28].

Neoadjuvant chemotherapy has recently become the standard of care for biologically high-risk breast cancers. Achieving pCR at the time of surgery is a surrogate marker for better long-term breast cancer outcomes [6, 29]. The Collaborative Trials in Neoadjuvant Breast Cancer (CTNeoBC) results indicated a long-term benefit for patients achieving pCR, as pCR was positively associated with overall EFS (hazard ratio 0.48, 95% CI 0.43–0.54) and overall OS (hazard ratio 0.36, 95% CI 0.31–0.42) [7]. Monitoring pCR rates among overweight and obese breast cancer patients who received neoadjuvant chemotherapy may help us understand why higher BMI is associated with poorer breast cancer outcomes.

Litton *et al* did the first large retrospective study in this regard, finding that patients with higher BMI were more likely to present with high-risk tumor characteristics and were less likely to achieve pCR after neoadjuvant chemotherapy; and that higher BMI was associated with worse OS [8]. Elsamany and colleagues performed a similar retrospective analysis in Saudi Arabian and Egyptian populations, and Fontanella *et al* did a pooled analysis of four clinical trials in Germany, both studies showed high BMI was associated with worse pCR rate [10, 11]. However, similar studies by Erbes *et al* and Kogawa *et al* did not reveal any statistically significant association between increased BMI and worse pCR [12, 14]. We previously performed a meta-analysis with total of 18,702 patients, with pooled univariable analysis demonstrating increased BMI was associated with worse pCR rate in overweight and obese patients [30]. Yet this meta-analysis has limitations given most included studies were retrospective in nature, multivariable analysis and subgroup analysis based on different subtypes of breast cancer were not able to be performed due to lack of standardization of patient characteristics; there were significant variations of chemotherapy regimens, and inclusion of non-weight based chemotherapy dosing [30].

Using the I-SPY 2 trial data to investigate the association of increased BMI with pCR outcome has several advantages. First, this is a currently active clinical trial platform using standard concurrent treatment regimens for each subtype of breast cancer, with a focus on treating high risk, biologically active breast cancer. Second, the I-SPY 2 trial uses standard treatment protocols and chemotherapy is given based on actual body weight. Lastly, it is one of the largest multicenter randomized clinical trials focusing on neoadjuvant therapy for breast cancer. These advantages may eliminate the potential biases originating from the variation of chemotherapy regimens and the underdosing of chemotherapy agents in patients with elevated BMI. In this strictly designed clinical trial, we did not identify any statistically significant evidence that higher BMI was associated with decreasing pCR rate in the high-risk early stage breast cancer group with various hormonal subtypes; nor within each hormonal subtype group after stratification. This result was different from most of the retrospective studies discussed above.

Our study reinforced the potential importance of dosing chemotherapy based on actual body weight. Some clinicians may reduce chemotherapy dosage in overweight and obese patients because of the fear of overdosing and excessive toxicity with higher chemotherapy dosage, although randomized clinical trials have demonstrated that this practice contributes to worse outcomes and guidelines recommend against this practice [31–33]. In the I-SPY 2 trial, chemotherapy dosing is strictly based on actual body weight, even if patients’ BSA is above 2.0m2. In Litton’s study, the chemotherapy dose of each patient was not documented and not able to be verified [8]. In Fontanella’s study, more than half of the study population had chemotherapy dosage capped at 2.0m2 [11]. It is possible that the poorer breast cancer outcomes in overweight and obese patients from these studies was attributable to chemotherapy underdosing rather than the influence of BMI on the chemotherapy effectiveness in these patients.

Our study has several limitations. Although the I-SPY 2 trial is a prospective study, the correlation of BMI to pCR is not the predetermined primary end point of this trial. While our analysis included almost 1000 women, dividing the study population by tumor subtype, ethnicity and BMI limited our statistical power; especially in the subgroup analysis of BMI in different breast cancer subtypes and its impact on breast cancer outcomes. As there were too few deaths/recurrences in patients who achieved pCR (RCB = 0), we were not able to run a meaningful survival analysis to determine whether BMI has an impact on OS and EFS regardless of patients’ pCR status. Longer follow up is needed to understand the overall impact on OS and EFS.

## Conclusion

We observed no difference in pCR rates by baseline BMI in this biologically high-risk breast cancer population receiving actual body weight-based neoadjuvant chemotherapy. These findings suggest the importance of treating overweight and obese patients with chemotherapy dosage based on actual weight. Longer follow up and further work, however, is needed to understand the role of body mass and breast cancer outcomes across all breast cancer subtypes.

## Figures and Tables

**Table 1:** Patient characteristics according to BMI category.

Patient Characteristics	BMI < 25 (N = 348)	25 ≤BMI < 30 (N=309)	BMI ≥30 (N=320)	Total (N=977)	P
	# of pts (%)	# of pts (%)	# of pts (%)	# of pts (%)	
**Age at Initial Treatment**					
Mean (STD)	46.8 (10.6)	49.9 (10.7)	49.7 (10.1)	48.7 (10.6)	**<.0001**
**Race/Ethnicity**					**<.0001**
African American / Black	21 (6)	26 (8.4)	71 (22.2)	118 (12.1)	
American Indian / Native or Hawaiian / Pacific Islander	4 (1.2)	2 (0.7)	5 (1.6)	11 (1.1)	
Asian	35 (10.1)	27 (8.7)	9 (2.8)	71 (7.3)	
Hispanic	23 (6.6)	43 (13.9)	49 (15.3)	115 (11.8)	
Non-Hispanic White	265 (76.2)	211 (68.3)	186 (58.1)	662 (67.8)	
**Menopausal Status**					0.14
Pre-	193 (64.3)	147 (57.0)	135 (54.4)	475 (58.9)	
Peri-	12 (4.0)	9 (3.5)	13 (5.2)	34 (4.2)	
Post-	95 (31.7)	102 (39.5)	100 (40.3)	297 (36.9)	
*Missing*	*48*	*51*	*72*	*171*	
**Hormonal and HER2 Status**					0.53
HR+/HER2+	63 (18.1)	44 (14.2)	48 (15.0)	155 (15.9)	
HR+/HER2-	130 (37.4)	130 (42.1)	118 (36.9)	378 (38.7)	
HR-/HER2+	28 (8.1)	31 (10.0)	29 (9.1)	88 (9.0)	
**Age at Initial Treatment**					
HR-/HER2-	127 (36.5)	104 (33.7)	125 (39.1)	356 (36.4)	
**Cancer Stage**					0.14
I	8 (2.7)	9 (3.5)	4 (1.6)	21 (2.6)	
II	222 (75.5)	173 (66.8)	183 (70.9)	578 (71.3)	
III	64 (21.8)	77 (29.7)	71 (27.5)	212 (26.1)	
*Missing*	*54*	*50*	*62*	*166*	

**Table 2:** Odds Ratios (OR) of pathological complete response (pCR) by BMI categories and adjusted variables

	# of pts	pCR	OR (95% CI)	P	OR (95% CI)	P
	(N)	N (%)	Unadjusted		Adjusted*	
**BMI**						
Normal / Underweight	348	114 (32.8)	1 (Ref.)		1 (Ref.)	
Overweight	309	97 (31.4)	0.94 (0.68–1.30)	0.71	0.97 (0.64– 1.47)	0.88
Obese	320	104 (32.5)	0.99 (0.71–1.37)	0.94	1.05 (0.68– 1.63)	0.83
**BMI (continuous, per additonal unit)**			0.99 (0.97–1.01)	0.50	0.99 (0.96– 1.02)	0.64
**Model stratified by cancer status (unadjusted odds ratios for continuous BMI, per additional unit)**						
**Subtype stratum**						
HR+/HER2−	378	64 (16.9)	1.01 (0.96–1.05)	0.81		
HR+/HER2+	155	57 (36.8)	0.97 (0.91–1.03)	0.32		
HR−/HER2+	88	55 (62.5)	0.93 (0.86–1.00)	0.06		
HR−/HER2−	356	139 (39.0)	1.00 (0.97–1.04)	0.99		

* Adjusted for age at screening, hormonal cancer subtype, race, stage, and menopausal status

**Table 3:** pCR rate of different BMI categories by breast cancer subtypes.

Breast Cancer Subtype	pCR	Normal/Underweight N (%)	Overweight N (%)	Obese N (%)	P
**HR+/HER2+**	**No**	39 (61.9)	27 (61.4)	32 (66.7)	0.83
	**Yes**	24 (38.1)	17 (38.6)	16 (33.3)	
**HR+/HER2-**	**No**	105 (80.8)	116 (89.2)	93 (78.8)	0.06
	**Yes**	25 (19.2)	14 (10.8)	25 (21.2)	
**HR-/HER2+**	**No**	7 (25.0)	11 (35.5)	15 (51.7)	0.11
	**Yes**	21 (75.0)	20 (64.5)	14 (48.3)	
**HR-/HER2-**	**No**	83 (65.4)	58 (55.8)	76 (60.8)	0.33
	**Yes**	44 (34.7)	46 (44.2)	49 (39.2)	

(HR: hormone receptor, HER2: human epidermal growth factor receptor 2, pCR: pathological complete response)

**Table 4:** Association of BMI categories and continuous RCB index in patients who did not achieve pCR.

	Coefficient (95% CI)	P	Coefficient (95% CI)	P
	Unadjusted		Adjusted*	
**BMI**		0.95		0.56
Normal / Underweight	0 (Ref.)		0 (Ref.)	
Overweight	0.02 (−0.20–0.24)	0.86	−0.14 (−0.38–0.11)	0.28
Obese	0.03 (−0.18–0.25)	0.75	−0.06 (−0.32–0.20)	0.67
**BMI (continuous, per additonal unit)**	0.00 (−0.01–0.02)	0.54	0.00 (−0.02–0.02)	0.84
**Model stratified by cancer status (coefficients for continuous BMI, per additional unit)**				
**Subtype stratum**				
HR+/HER2−	0.00 (−0.02–0.02)	0.87		
HR+/HER2+	0.01 (−0.03–0.05)	0.61		
HR−/HER2+	0.03 (−0.01–0.07)	0.13		
HR−/HER2−	0.00 (−0.02–0.03)	0.80		

* Adjusted for age at screening, hormonal cancer subtype, race, stage, and menopausal status

**Table 5:** Association of BMI with overall survival (hazard ratios, HR)

BMI	Adjusted HR*	95% CI	P
Normal / Underweight	1 (Ref.)		
Overweight	1.13	0.69–1.87	0.63
Obese	0.82	0.46–1.44	0.48

* Adjusted for age, hormonal cancer subtype, stage, and menopausal status

**Table 6:** Association of BMI with Event-free Survival

BMI	Adjusted HR*	95% CI	P
Normal / Underweight	1 (Ref.)		
Overweight	0.99	0.67–1.49	0.98
Obese	0.88	0.57–1.35	0.56

* Adjusted for age, hormonal cancer subtype, stage, and menopausal status

## Data Availability

Data is available upon request.

## Abbreviations

BMI: Increased body mass index
pCR: Pathological complete response
EFS: Event-free survival
OS: Overall survival
BSA: Body-surface area
I-SPY 2: Investigation of Serial studies to Predict Your Therapeutic Response with Imaging and Molecular AnaLysis 2
HR: Hormone receptors
HER2: Human epidermal growth factor receptor 2
RCB: Residual cancer burden
OR: Odds ratios
CI: Confidence intervals
